# Caecal metastasis from breast cancer presenting as intestinal obstruction

**DOI:** 10.1186/1477-7819-6-47

**Published:** 2008-05-09

**Authors:** Rashmi Birla, Kamal Kumar Mahawar, Mavis Orizu, Muhammad S Siddiqui, Arun Batra

**Affiliations:** 1 Department of General Surgery, University Hospital of Hartlepool, Hartlepool, TS24 9AH, UK; 2 Department Of Pathology, University Hospital of Hartlepool, Hartlepool, TS24 9AH, UK; 3 Department Of Radiology, University Hospital of Hartlepool, Hartlepool, TS24 9AH, UK

## Abstract

**Background:**

Gastrointestinal metastsasis from the breast cancer are rare. We report a patient who presented with intestinal obstruction due to solitary caecal metastasis from infiltrating ductal carcinoma of breast. We also review the available literature briefly.

**Case presentation:**

A 72 year old lady with past history of breast cancer presented with intestinal obstruction due to a caecal mass. She underwent an emergency right hemicolectomy. The histological examination of the right hemicolectomy specimen revealed an adenocarcinoma in caecum staining positive for Cytokeratin 7 and Carcinoembryonic antigen and negative for Cytokeratin 20, CDX2 and Estrogen receptor. Eight out of 11 mesenteric nodes showed tumour deposits. A histological diagnosis of metastatic breast carcinoma was given.

**Conclusion:**

To the best of our knowledge, this is the first case report of solitary metastasis to caecum from infiltrating ductal carcinoma of breast. Awareness of this possibility will aid in appropriate management of such patients.

## Background

Metastasis from the breast cancer to the gastrointestinal tract is rare. Presentation of such patients can mimic that of primary bowel neoplasm and the exact diagnosis is often only made on detailed immunohistochemical study. Appropriate management requires the condition to be kept in mind while dealing with such cases. We report a lady who presented with intestinal obstruction due to solitary caecal metastasis from infiltrating ductal carcinoma of breast. We also review the available literature briefly.

## Case presentation

A 72 year old lady presented to us as an emergency with abdominal pain, intermittent vomiting and worsening constipation of a few days duration. She also reported a significant weight loss over past few months. Her relevant past history included rheumatoid arthritis and pT1 N0 M0 carcinoma of the right breast, 3 years ago, for which she underwent wide local excision and axillary node sampling followed by adjuvant radiotherapy. She was also on Arimidex as hormonal therapy. Her general examination was unremarkable and the abdominal examination revealed a distended abdomen with a suggestion of fullness in the right iliac fossa.

**Figure 1:**
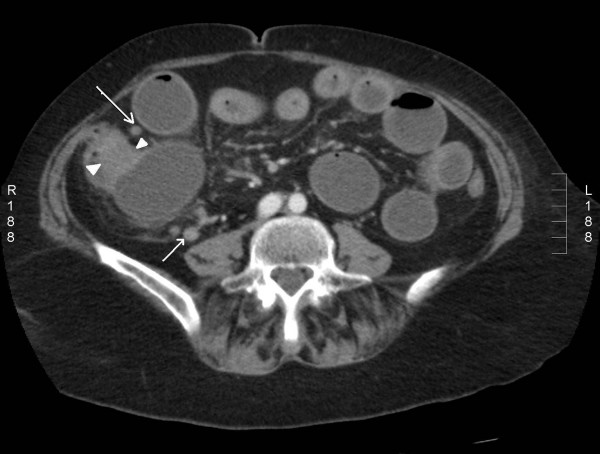
An axial CT image showing dilated small bowel loops and concentric thickening of caecal wall (arrow heads) close to the ileocaecal junction. Pericolic and ileocolic lymphnodes are also seen (arrow).

A computed tomography (CT) scan of the abdomen showed a caecal mass causing intestinal obstruction (figure 1). The patient underwent an emergency right hemicolectomy and made a satisfactory postoperative recovery.

At 13 months follow up she had no signs of recurrence of tumour. CT Scan of her chest and abdomen did not show any visceral metastasis. A Magnetic Resonance Imaging Scan and Bone Scan with intravenous MBq Tc 99m-HDP with imaging at 3 hours ruled out bony metastasis. Carcinoembryonic Antigen (CEA) and Cancer Antigen 15-3 (CA153) levels done 6 monthly in the follow up period were within normal limits.

**Figure 2:**
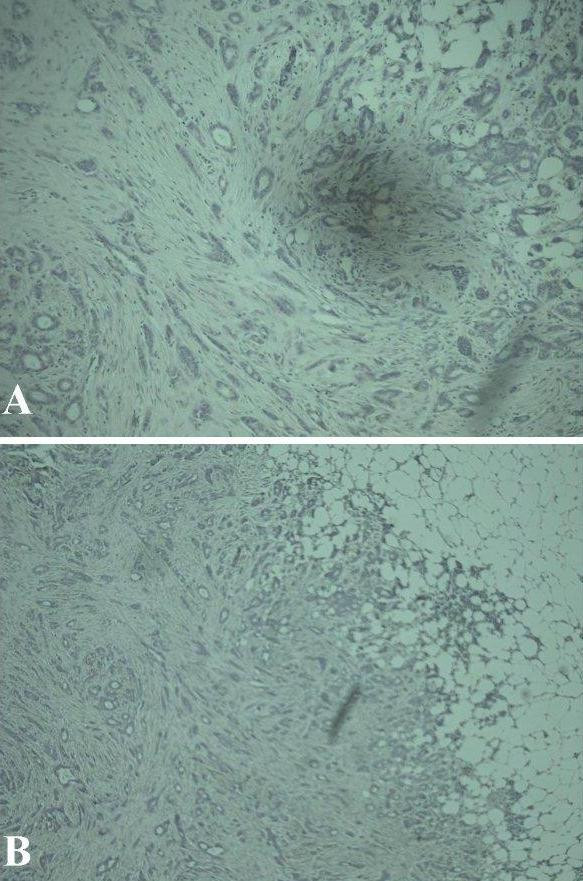
Photomicrograph A) and B): Primary breast infiltrating ductal carcinoma.

The histology of the wide local excision and axillary sampling specimen had revealed a grade 1 infiltrating ductal carcinoma (Figure 2A and 2B) with no lymphovascular invasion. The tumour was 11 mm in maximum diameter and the closest radial margin was 6 mm inferiorly. None of the thirteen lymph nodes recovered showed any evidence of metastasis. It was positive for both Estrogen and Progesterone receptors. Expression of HER 2 protein was negative. There was only focal ductal carcinoma in situ (DCIS) seen within the tumour.

**Figure 3:**
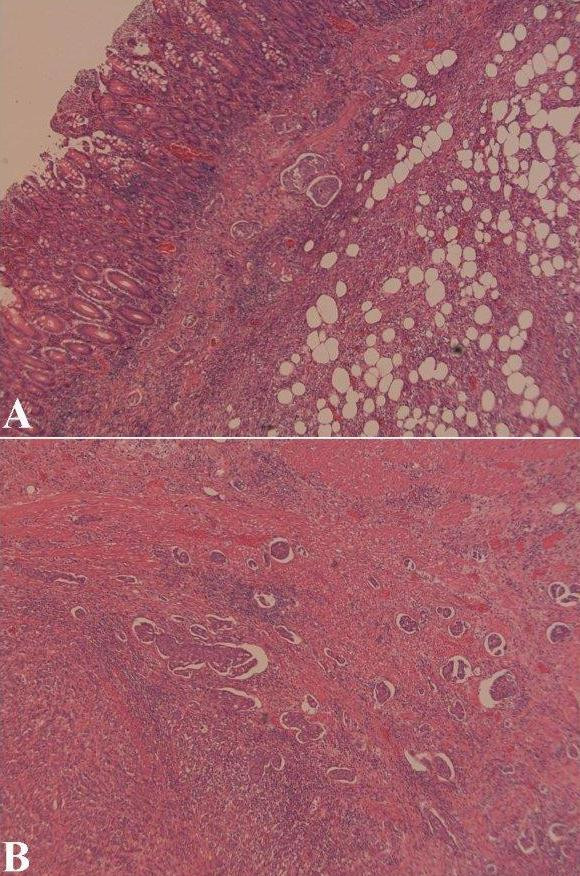
Photomicrograph A) H and E stained slide showing tumour groups in the lamina propria, muscle and fat covered by benign colonic mucosa, **B) **Tumour cell groups in the wall and in the vessels.

**Figure 4:**
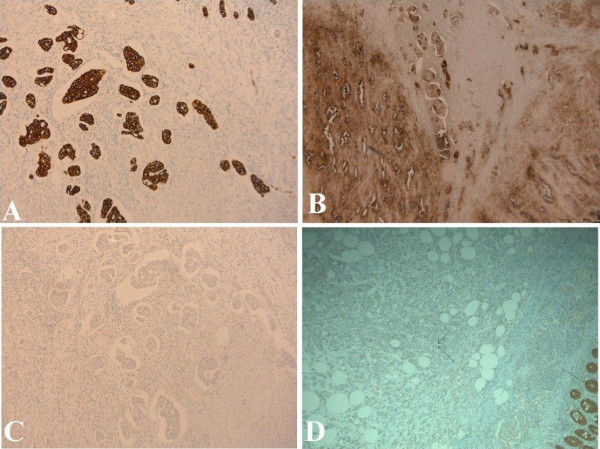
photomicrograph **A) **Immunostaining showing CK7 positivity in tumour cell groups. **B): **Immunostaining showing CEA positivity in tumour cell groups. **C): **Immunostaining showing CK20 negativity in tumour cell groups. **D): **Immunostaining for CDX2. There is positive staining in normal colonic mucosa (single arrow) whereas the tumour beneath the mucosa stains negative (two arrows).

On histopathological examination of the right hemicolectomy specimen, an ulcerated tumour was identified in the caecum. Multiple sections from the caecum showed an adenocarcinoma with tumour cells in nests and groups with focal cribriform pattern. The tumour extended into the mucosa, muscle and the subserosa. No transformation to malignant epithelium was identified in multiple sections (figure 3A and 3B). Proximal and distal resection margins were tumour free. Immunohistochemistry showed positive staining with Cytokeratin (CK) 7 (figure 4A) and CEA (figure 4B) whereas staining with CK20 (figure 4C), CDX2 (figure 4D) and Estrogen receptor(ER) were negative. Progesterone receptor (PR) showed equivocal nuclear staining. Eight out of eleven mesenteric nodes showed tumour deposits. A histological diagnosis of metastatic breast carcinoma was made in light of the histological pattern of the tumour, previous history of breast cancer, positive immunostaining with CK7 and CEA and negative with CK20 and CDX2.

## Discussion

Breast cancer is the commonest cancer in females in the western population. Common sites of metastasis are lymph nodes, bone, lungs, liver, brain and skin. Metastasis to the gastro intestinal tract, though very rare is known, and may require surgical intervention [1,2]. In an autopsy study of 707 patients by Cifuentes and Pickren [3] metastases to the gastrointestinal tract were detected in 16% cases with breast carcinoma (stomach 10%, small intestine 9%, and large intestine 8%). There have been isolated case reports of metastasis to rectum [4] and ileocaecal valve [5].

Although, breast cancer metastases to gastrointestinal tract usually arise from lobular variety and are usually disseminated on presentation, solitary metastasis from ductal carcinoma to the ileocecal valve is reported [5]. Wai Lun Law *et al *[6] have also described a case of scirrhous colonic metastasis, infiltrative in nature from ductal carcinoma of the breast. However, to the best of our knowledge, this is the only report of solitary metastasis to the caecum from infiltrating ductal carcinoma of the breast.

Patients with a history of breast cancer presenting with anaemia and/or bowel obstruction should be investigated for possible metastasis to bowel [7].

Establishing the histological origin of adenocarcinoma i.e. primary or metastatic however can be challenging. There isn't any single marker available to aid in determining the primary site in cases of metastatic adenocarcinomas, and therefore a combination of markers is often employed.

Metastatic breast cancers are usually positive for CK 7, CEA, ER, PR and gross cystic disease fluid protein 15 (GCDFP 15) [2,8]. CK 7 and CEA positivity is non-specific [5]. However, CK 20 is almost invariably present in gastrointestinal tumours and absent in breast carcinomas [5,9]. JH Lagendijk *et al *[10] have also observed in their study that although the immunostaining patterns show a considerable overlap, the breast carcinomas were typically positive for GCDFP-15 and often for ER, and negative for vimentin whereas colonic carcinomas showed prominent positivity for CEA and CK20, while no staining was seen for ER and vimentin.

Seog-Yun Park *et al *[11] have recently proposed a decision tree and a design of multiple-marker panels using 10 markers (CDX2, CK7, CK20, thyroid transcription factor 1 (TTF-1), CEA, MUC2, MUC5AC, SMAD4, ER, GCDFP-15) to determine the origin from seven primary sites (colon, stomach, lung, pancreas, bile duct, breast, ovaries). In their study, they found the immunostaining profile for the origin of metastatic adenocarcinomas from the breast to be GCDFP-15+/TTF-1-/CDX2-/CK7+/CK20- or ER+/TTF-1-/CDX2-/CK20-/CEA-/MUC5AC- and that of colorectal origin to be TTF-1-/CDX2+/CK7-/CK20+ or TTF-1-/CDX2+/CK7-/CK20-/(CEA+ or MUC2+).

In an interesting case report by Santini D *et al*, an increase in Cancer Antigen (CA) 19.9 was used to diagnose ileocaecal valve metastasis from breast cancer in an otherwise asymptomatic patient [12].

Hence positive staining for CK 7 and negative staining for CK 20 and CDX2 in our patient favours a diagnosis of metastatic breast carcinoma [2,5,8,9,11].

The original breast cancer was positive for both ER and PR. The histopathological specimen of caecal tumour after right hemicolectomy stained negative for ER and equivocal for PR. Such discordance in hormone receptor status between primary and metastatic breast cancer lesions has been noted by other authors [13,14] previously. Lower EE *et al *[13] noticed a higher incidence of discordance with distant metastasis compared to local recurrence.

Heterogeneity in receptor status within a tumour mass has also been described [15]. There is no consensus on possible causes but endocrine treatment, variations in tissue sampling and technical difficulty have been suggested for the discordance in the receptor status [13,16].

It is important to be aware of the possibility of gastrointestinal metastasis from breast as the management may be different from a primary bowel neoplasm. Metastatic breast cancer with intestinal involvement may warrant systemic hormonal or chemotherapy either alone or combined with surgery [17]. In our case, we did not suspect the lesion to be a caecal metastasis from breast until indicated by histopathology. Also, since the patient was obstructed, she needed the surgery on emergency basis. Both these factors precluded any possible preoperative systemic anti cancer treatment in this patient. An initial attempt at postoperative adjuvant chemotherapy also had to be quickly abandoned due to poor patient tolerance.

Bowel surgery in post mastectomy patients who have undergone Transverse Rectus Abdominis Myocutaneous (TRAM) flap would need careful preoperative planning of surgical incision and any possible stoma [6].

There have been interesting case reports in literature, of metastatic breast cancer presenting with bowel perforation in patients receiving chemotherapy [18,19] as well as those not receiving chemotherapy [20]. Daniel A *et al *[21] have reported a case of oesophageal perforation in a patient with oesophageal metastasis from breast. Careful evaluation of gastrointestinal tract in patients with advanced breast cancer receiving chemotherapy may prevent intestinal perforation [19].

## Conclusion

Gastrointestinal metastasis from breast carcinoma may mimic primary bowel neoplasm in presentation. Immunohistochemistry may aid in differentiating between the two conditions. Accurate diagnosis will help in formulating a proper management plan. Surgeons should bear this condition in mind while treating patients with a past history of breast cancer presenting with bowel obstruction.

## List of abbreviations

CK: Cytokeratin; CEA: Carcinoembryonic antigen; ER: Estrogen receptor; PR: Progesterone receptor; GCDFP: Gross cystic disease fluid protein; CA: Cancer antigen; DCIS: Ductal carcinoma in situ; CT: Computed tomography; MRI: Magnetic Resonance Imaging; TRAM: Transverse Rectus Abdominis Myocutaneous

## Competing interests

The authors declare that they have no competing interests.

## Authors' contributions

**RB **reviewed the literature and wrote the manuscript. **KM **conceived the case report and helped with writing of the manuscript. **MO **helped in collecting the images. **MS **was pathologist on the case, and helped with pathological sections in the manuscript. **AB **helped with the radiological images. All authors read the manuscript and agreed with it.
